# Commentary on “The number of undocumented immigrants in the United States: Estimates based on demographic modeling with data from 1990-2016”

**DOI:** 10.1371/journal.pone.0204199

**Published:** 2018-09-21

**Authors:** Randy Capps, Julia Gelatt, Jennifer Van Hook, Michael Fix

**Affiliations:** 1 Migration Policy Institute, Washington, D.C., United States of America; 2 Population Research Institute and Departments of Demography and Sociology, The Pennsylvania State University, State College, Pennsylvania, United States of America; Public Library of Science, UNITED KINGDOM

## Abstract

“The number of undocumented immigrants in the United States: Estimates based on demographic modeling with data from 1990–2016” by Fazel-Zarandi, Feinstein and Kaplan presents strikingly higher estimates of the unauthorized immigrant population than established estimates using the residual method. Fazel-Zarandi et. al.’s estimates range from a low or “conservative” number of 16.7 million unauthorized immigrants, to an “average” of 22.1 million, and to a high of 27.5 million. The Pew Hispanic Center estimated the population at 11.3 million in 2016, and the Department of Homeland Security (DHS) estimated it at 12.3 million. The new method shows much more rapid growth in unauthorized immigration during the 1990s and a substantially higher population in 2000 (13.3 million according to their “conservative” model) than Pew (8.6 million) and DHS (8.5 million). In this commentary, we explain that such an estimate for 2000 is implausible, as it suggests that the 2000 Census undercounted the unauthorized immigrant population by at least 42% in the 2000 Census, and it is misaligned with other demographic data. Fazel-Zarandi, Feinstein and Kaplan’s model produces estimates that have a 10 million-person range in 2016, far too wide to be useful for public policy purposes; their estimates are not benchmarked against any external data sources; and their model appears to be driven by assumptions about return migration of unauthorized immigrants during the 1990s. Using emigration rates from the binational Mexican Migration Project survey for the illegal border-crosser portion of the unauthorized population, we generate a 2000 unauthorized population estimate of 8.2 million—slightly below Pew and DHS’s estimates—without changing other assumptions in the model. We conclude that this new model’s estimates are highly sensitive to assumptions about emigration, and moreover, that the knowledge base about emigration in the unauthorized population during the 1990s is not well enough developed to support the model underlying their estimates.

## Introduction

Accurately estimating the U.S. unauthorized immigrant population—the population that entered the country illegally, usually across the border with Mexico, or that overstayed a valid visa—is critical to informing border security, immigration enforcement, and immigrant admissions policies that are vital to the health of the nation. For decades, demographers have estimated the size of the unauthorized population using a residual method. This method involves subtracting the known legal immigrant population—calculated based on administrative records of immigrant and nonimmigrant admissions—from the total foreign-born population, obtained from Census Bureau surveys: the decennial Census in earlier years and most recently the annual American Community Survey (ACS) [1, 2].

In their article, “The Number of Undocumented Immigrants in the United States: Estimates Based on Demographic Modeling with Data from 1990 to 2016,” Mohammad M. Fazel-Zarandi, Jonathan S. Feinstein, and Edward H. Kaplan present an alternative method for estimating the unauthorized population [3]. Instead of subtracting the legal immigrant population from the total foreign-born population in the Census and ACS, the authors begin with a widely accepted starting point of 3.5 million unauthorized immigrants in 1990, and project the population forward to 2016 by adding the estimated number of migrants crossing the border illegally and overstaying valid visas each year, minus estimated emigrants, deaths, and adjustments to legal status. Their method employs new data from the U.S. Department of Homeland Security (DHS) on estimated illegal border crossings and visa overstays for recent years—data unavailable when demographers developed the residual method.

Fazel-Zarandi, Feinstein and Kaplan’s methodology makes sense in principle. It accounts for all of the ways that unauthorized immigrants are added or subtracted from the population; it makes creative use of new data on visa overstays and unauthorized border crossings; and it accounts for uncertainty by producing a range of estimates based on a range of underlying assumptions.

However, for several reasons that we elaborate below, Fazel-Zarandi, Feinstein and Kaplan’s approach is very sensitive to underlying assumptions. As a consequence, their estimates range widely and are too uncertain to be of value for policy purposes. Additionally, the knowledge base about key inputs for their model—particularly assumptions about the circularity of unauthorized migration flows during the 1990s—is insufficiently developed to support their approach. As we discuss below, it is very likely that Fazel-Zarandi, Feinstein and Kaplan’s estimates of growth during the 1990s are too high because of the assumptions used about the level of return migration among illegal border crossers during the 1990s. Because of the way their estimates build over time, this causes their estimates to expand rapidly, compounding errors over the years and yielding much higher estimates than those implied by alternative indicators of the size of the unauthorized population. While their new method has the potential to move the field forward, it must be informed by accurate data and assumptions, and must be benchmarked against information external to their model. As it is, their model generates results that are inconsistent with estimates based on other data sources—most notably the U.S. and Mexican Censuses—while not being adequately grounded in empirical research about patterns of unauthorized migration, particularly for Mexican border crossers during the 1990s.

In this commentary, we first describe the key assumptions underlying the different estimation methods. Next, we critically evaluate Fazel-Zarandi, Feinstein and Kaplan’s estimates and the assumptions driving these estimates during the period between 1990 and 2000 because it is during this period that their estimates diverge most from prior estimates. Finally, we produce alternative estimates of growth to demonstrate how sensitive Fazel-Zarandi, Feinstein and Kaplan’s estimates are to assumptions of emigration during the 1990s.

### Assumptions in the estimation methodologies

Both methods rely on key assumptions. The residual method involves adding up the number of people obtaining temporary visas or green cards each year, and then reducing this legal immigrant population based on mortality and emigration over time. DHS data on legal immigrant admissions and adjustments are thorough and detailed, but mortality and emigration rates must be estimated based on studies of other populations. Next, when subtracting the legal immigrant population from the total foreign-born population appearing in the Census and ACS, demographers must factor in assumptions about the share of immigrants in the United States who were captured in the Census or ACS data. Estimates of undercounts generated from small surveys range from 10% in the 2000 Census to as high as 20% in 2010 [4,5]. Unauthorized immigrants are frequently low-income, move more often than average U.S. residents, and are often seeking to avoid contact with public authorities, making them less likely to respond to Census Bureau surveys than other populations.

The potential advantage of the authors’ new method is that it avoids the necessity of assuming an undercount rate in the Census or ACS, thereby eliminating one source of uncertainty. But the new method also relies heavily on many other assumptions. To their credit, the authors provide a range of estimates that correspond with a range of assumptions about the model inputs. Unfortunately, however, their estimates range widely. These estimates are too uncertain to be of value for policy purposes. Residual estimates, in contrast, tend to cluster more tightly. The estimates produced by Pew, DHS, and CMS often fall within half a million of each other. We concede that it would be helpful to develop uncertainty ranges around residual estimates, but estimates that could range anywhere from 17 million to 27 million (based on the range between Fazel-Zarandi, Feinstein and Kaplan’s conservative estimate of 17 million and their average estimate of 22 million plus 5 million) are limited for purposes of social science and public policy evaluation. (The authors provide a line chart that includes an even broader range from 12 million to 37 million.) They would provide broad ranges of estimates (in the millions) for bills legalizing a portion of the unauthorized such as those with at least 10 years of U.S. residence. It would also be difficult to estimate with any precision the number of people eligible for Deferred Action for Childhood Arrivals (DACA) or the economic impacts of unauthorized immigrants on states and local communities.

Another limitation to Fazel-Zarandi, Feinstein and Kaplan’s method is that the errors accumulate over time. Because their estimate of the unauthorized foreign-born population is built up from year to year by adding and subtracting entries and exits from the population, errors in earlier years are carried forward into later years. This does not pose a problem if the error is randomly distributed (i.e., producing a lower estimate in some years and higher in others). However, if the error is systematic, then what may appear to be a very small error during the early years of the estimation period can accumulate to a much larger error in later years. Fazel-Zarandi, Feinstein and Kaplan’s method does not build in any comparisons with external data sources such as a U.S. or Mexican Census, demographic estimate, or other benchmark, and the lack of benchmarks allows their estimates to increasingly diverge from others’ over time. In contrast, the residual method is partially recalibrated each time a new census is conducted. Each new census provides an updated, independent measure of the size of the foreign-born population, and importantly, the errors of earlier censuses do not have any direct or cumulative effect on the new estimates. This is similar to when the Census recalibrates its population estimates every ten years using decennial census counts; these recalibrations are then used to weight the ACS, Current Population Survey and other data sources that the public uses.

In what follows, we provide an example of how inaccurate assumptions during the earlier years can throw off Fazel-Zarandi, Feinstein and Kaplan’s estimates for later years.

### Divergence in estimates for 2000

We start by noting that even though Fazel-Zarandi, Feinstein and Kaplan’s estimates of the total unauthorized population are much higher than residual estimates, their estimates of *growth* after 2000 are actually quite consistent with estimates of *growth* implied by residual estimates for the same time period. This means that the discrepancy between their 2016 unauthorized population estimate and others’ estimates can be attributed primarily to differences in estimates of growth of the population during the 1990s.

As a starting point for their analysis, Fazel-Zarandi, Feinstein and Kaplan use the commonly accepted estimate that there were 3.5 million unauthorized immigrants in the U.S. in 1990. This estimate was developed using the residual method, which became widely accepted after the US Immigration and Naturalization Service (INS) used the method to estimate the number of unauthorized immigrants eligible to legalize their status under the terms of the 1986 Immigration Reform and Control Act (IRCA). The INS projected an eligible population of between 1.3 and 2.6 million, and ultimately 1.6 million unauthorized immigrants came forward to legalize [6].

However, Fazel-Zarandi, Feinstein and Kaplan’s estimate of the unauthorized immigrant population in 2000 diverges considerably from leading estimates using the residual method: those by DHS [7] and the Pew Research Center [8]. According to what Fazel-Zarandi, Feinstein and Kaplan characterize as their “conservative” estimate, the unauthorized population grew by 9.8 million during the 1990s, from 3.5 million to 13.3 million (see Table 1). Over that decade, the estimated unauthorized population rose only about half as much using the residual method: by 5.1 million according to Pew and 5.0 million according to DHS.

**Table 1:** Unauthorized population estimates for 1990, 2000, and 2014.

	1990	2000	2014	Change 1990 to 2000	Change 2000 to 2014
**New method** Fazel-Zarandi, Feinstein and Kaplan (conservative)	3.5 million	13.3 million	16.5 million	9.8 million	3.2 million
**Residual method** Pew Research Center	3.5 million	8.6 million	11.1 million	5.1 million	2.6 million
Department of Homeland Security	3.5 million	8.5 million	12.1 million	5.0 million	3.6 million

There is not much difference among the different methods’ estimates of growth in the unauthorized population after 2000. Using Fazel-Zarandi, Feinstein and Kaplan’s conservative estimates, the unauthorized population grew by 3.2 million: from 13.3 to 16.5 million in 2014. The growth they estimate over the 2000–2014 period falls between estimates using the residual method: 2.6 million for Pew and 3.6 million for DHS. (Fazel-Zarandi, Feinstein and Kaplan’s conservative estimate rises slightly to 16.6 million for 2016, while Pew’s estimate rises to 11.3 million for that year. The latest estimate DHS provides is for 2014.) Thus, almost all variation in estimates of the 2014 unauthorized population between Fazel-Zarandi, Feinstein and Kaplan’s method and the residual method owes to divergences before 2000.

### 2000 estimate misaligned with data from the U.S. and Mexican Censuses

The results of Fazel-Zarandi, Feinstein and Kaplan’s models imply an implausibly high undercount of unauthorized immigrants in the 2000 U.S. Census. Their conservative estimate for 2000 is nearly 50% higher than estimates using residual models (13.3 million versus 8.5 to 8.6 million). Their “average” estimate for 2000 is even higher, nearly 20 million—or more than double the residual estimates, implying a huge undercount of unauthorized immigrants in the 2000 Census. The residual estimates already incorporate undercounts of unauthorized immigrants in the Census or ACS data: for example, the DHS estimate for 2014 includes 10.9 million unauthorized immigrants counted in the ACS plus 1.2 million who were not counted, for a total of 12.1 million and an undercount rate of 11% [7]. Considering that the 2000 U.S. Census included roughly 7.7 million unauthorized immigrants (implied by DHS estimate for 2000 and an 11-percent undercount), the Fazel-Zarandi, Feinstein and Kaplan conservative estimates imply that the Census undercounted the unauthorized population by 42% ((13.3–7.7)/13.3), and their average estimates imply an undercount of 62% ((20–7.6)/20). In other words, their estimates imply that the Census missed 4.7 to 16.5 million more unauthorized immigrants than previously assumed. Fazel-Zarandi, Feinstein and Kaplan’s 2010 estimates imply coverage error rates that are nearly as high, ranging from 35% in their conservative estimate to 53% in their average estimate.

Such high undercounts are implausible, even for the unauthorized population. In-depth research on immigrant populations following the 2000 U.S. Census suggested that only 10% of unauthorized Mexican migrants in Los Angeles said they did not answer the Census questionnaire [4]. Another study [9] estimated U.S. Census coverage error by estimating the size of the Mexican foreign-born population from independent sources that are not likely to undercount this group, namely birth and death records. The study also analyzed changes in Mexican Census data to gauge the size of Mexico’s “missing” population, most of whom moved to the United States. The study concluded that the 2000 U.S. Census missed about 15% of the entire Mexican-born population and no more than 26% of Mexican-born unauthorized immigrants. Moreover, by 2010, coverage error for the entire Mexican-born population declined to about 4% and between 5% and 7% among the unauthorized, far below the levels implied by Fazel-Zarandi, Feinstein and Kaplan’s estimates.

To better understand how coverage error could be so low, it is important to consider that the 2000 Census was successful in reaching the vast majority of American households (99.6% in 2000 according to official reports) [10]. The Census Bureau deploys a large staff to conduct a thorough survey of housing units in the United States, and thus misses very few units. For so many immigrants to be missed, the Census Bureau would have had to either misidentified millions of housing units as vacant or missed millions of people “doubled up” within surveyed households. Since the Census Bureau visited every unit perceived to be vacant at least once, and immigrant households were observed to be more densely occupied than those of natives, it seems highly implausible that the Bureau could have missed 4.7 to 16.5 million people in 2010.

### Estimating emigration of unauthorized migrants during the 1990s

What can account for the divergence in unauthorized population estimates between the Fazel-Zarandi, Feinstein and Kaplan method and the residual method during the 1990s? The 1990s was a time when circular unauthorized migration (back and forth between Mexico and the United States) and multiple crossings by the same individual within a year or two were common. The Fazel-Zarandi, Feinstein and Kaplan estimates depend on accurate counts of all entries and exits of unauthorized immigrants; if the estimated number of entries is correct but the corresponding estimate of exits is too low, population growth will be overestimated. The extent to which multiple crossings and return migration are factored into the assumptions underlying these methodologies therefore can substantially influence the results.

During the 1990s and early 2000s, the vast majority of border crossers were Mexican [11]. In 2000, at the end of the decade, just 2% of U.S. Border Patrol apprehensions (40,000 out of 1,676,000) were migrants from countries other than Mexico. Many unauthorized Mexican migrants came to the United States for periods of months or years to work, and then returned. Illegal borders crossers faced few consequences if they were caught, and most were returned to Mexico quickly and tried to reenter illegally again. Bi-national surveys of migrants in Mexico and the United States reported very high rates of circular migration during the 1990s [12, 13, 14] before the border between the two countries was fortified. Starting with Operation Hold the Line in El Paso in 1993, the Border Patrol strengthened enforcement in more urbanized areas along the U.S.-Mexico border by deploying more officers, physical barriers, and technology to monitor border crossings. By the end of the 1990s it was substantially more difficult to cross than it had been earlier in the decade [13, 14]. After 2000, the Border Patrol began imposing “consequences” on apprehended migrants such as bars on legal admission, prosecution and time in federal prisons, and repatriation through remote, often dangerous entry points along the border [15]. By 2005, increased border enforcement had led to a quadrupling of fees paid to smugglers since 1993, while substantially increasing the amount of time that unauthorized immigrants spent in the United States. Experts concluded that the new enforcement policies had broken the prior pattern of circular migration [13, 14].

Given that circular and repeat migration was much more common during the 1990s than afterwards, models that estimate growth in the illegal border crosser population based on data after 2000 likely do not apply to the earlier period. In particular, we focus on emigration rates for Mexican border crossers during the 1990s. We believe actual emigration rates during this period were higher than the rates incorporated in the models used by Fazel-Zarandi, Feinstein and Kaplan, and that their underestimation of emigration of border crossers during the 1990s is the primary reason for their much higher estimate of the total unauthorized population in 2000 than estimates reported by Pew and DHS. Part of the problem is that the emigration rates used by Fazel-Zarandi, Feinstein and Kaplan were estimated for the entire foreign-born population (total or from specific regions), and do not pertain specifically to undocumented border crossers during the 1990s, a group with much higher rates of return and circular migration. To obtain alternative emigration rates for the 1990s, we use data from the Mexican Migration Project (MMP), a binational study led by researchers at Princeton University and the University of Guadalajara in Mexico that tracked the character and behavior of documented and undocumented migration from Mexico to the United States starting in 1982 [16]. The MMP is an important and well-regarded source of information about circular migration patterns of undocumented border crossers from Mexico [17]. The Russell Sage Foundation, for instance, describes the MMP as “the largest, most comprehensive, and reliable source of data on Mexican immigrants currently available”.

Our alternative emigration rates for Mexican unauthorized migrants were developed from reported returns to Mexico among households that MMP sampled in Mexico and the United States. Between 1982 and 2017, MMP’s annual surveys interviewed over 27,000 households in 161 Mexican communities and over 1,000 households of Mexican immigrants in the United States [16]. The MMP data make it possible to calculate rates of emigration to Mexico for householders—generally adult males who were most likely to migrate—and for all household members, including children of the head of household not presently residing with their parents. The emigration rate to Mexico during migrants’ first year in the United States that Fazel-Zarandi, Feinstein and Kaplan employ (40%) is similar to the MMP rate for all household members during the 1990s (42%). However, the MMP data revealed much higher rates of emigration among Mexican immigrants who had been in the U.S. for longer periods of time than in the data employed by Fazel-Zarandi, Feinstein and Kaplan (see Table 2). For example, the emigration rate for those in the U.S. from 2 to 10 years was 20% for householders and 19% for all household members in the MMP, as opposed to just 4% in Fazel-Zarandi, Feinstein and Kaplan’s model. This is because the MMP captures the circular migration patterns of all trips taken by unauthorized border crossers, including very short trips. A review of data sources and methods for estimating emigration of immigrants from the U.S. by demographers concludes that other sources besides the MMP do not capture the dynamics of short trips [18]. It is important to account for return migration for all trips across the border because Fazel-Zarandi, Feinstein and Kaplan’s model counts all trips as entries—both those of circular migrants and more permanent U.S. residents.

**Table 2:** Emigration rates for border crossers during the 1990s.

	First year in the U.S.	In the U.S. 2–10 years	In the U.S. 11 or more years
**Fazel-Zarandi, Feinstein and Kaplan**	40%	4%	1%
**Mexican Migration Project** Householders only	50.2%	20.2%	16.6%
All household members	41.6%	19.4%	15.4%

**Fig 1:**
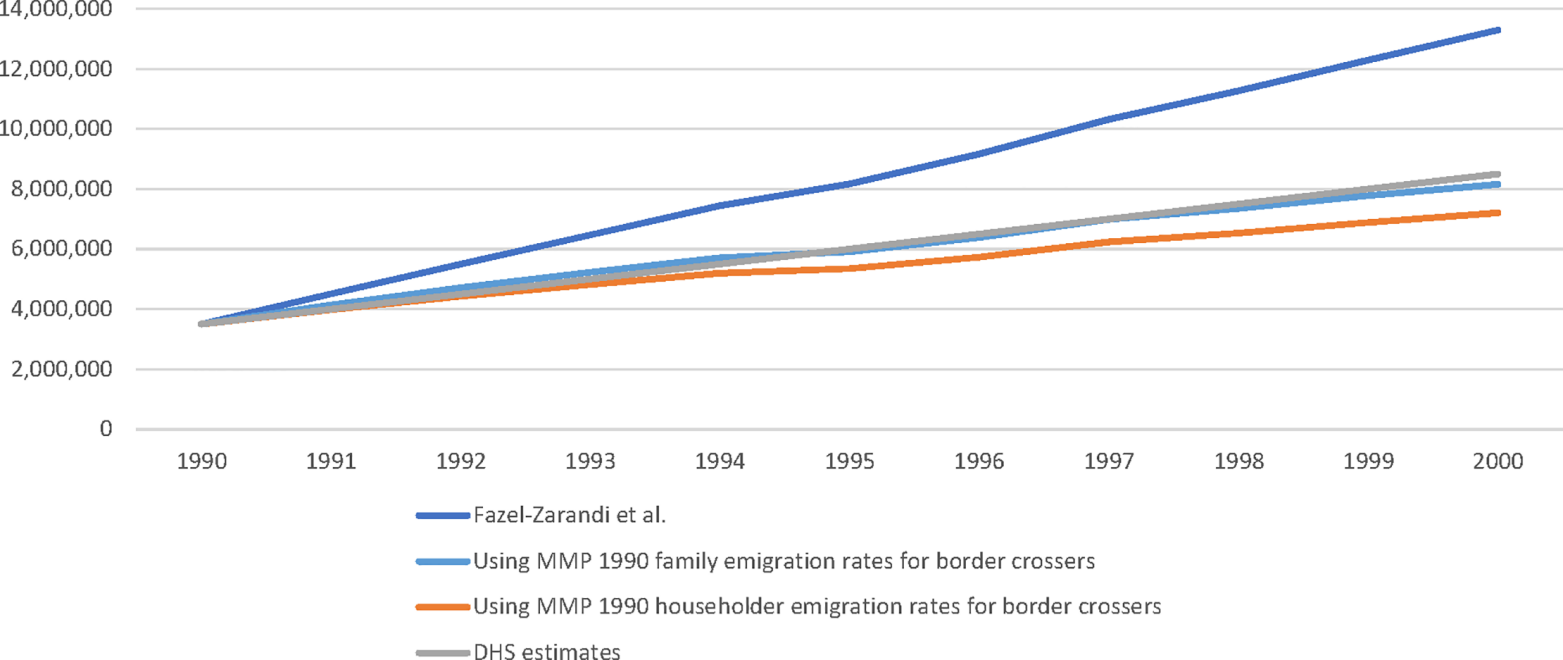
Estimated unauthorized populations, 1990 through 2000, using different emigration rates.

Changing the emigration rates for border crossers substantially alters the total unauthorized population estimates in the Fazel-Zarandi, Feinstein and Kaplan model. Using the emigration rates employed by the authors, the unauthorized immigrant population rises rapidly from 3.5 million in 1990 to 13.3 million in 2000 (see Fig 1). But if we use the higher MMP emigration rates for border crossers (while continuing to use Fazel-Zarandi, Feinstein and Kaplan’s rates for the visa overstay population), the total unauthorized population only increases to 8.2 million in 2000 (using the emigration rate for all household members) or to 7.2 million (using the rate for householders only). These estimates are slightly below the DHS estimate of 8.5 million, assuming that 62% of unauthorized immigrants in 1990 were border crossers, and 38% were visa overstays [19]. We only applied the MMP emigration rates to border crossers, while we applied the same emigration rates as Fazel-Zarandi, Feinstein and Kaplan to the overstay population.

We conducted this exercise to demonstrate how sensitive the estimates are to assumptions about emigration during the 1990s. Yet are the MMP emigration estimates plausible? If emigration is as high as 40% in the first year, 19.4% in years 2 through 10, and 15.4% thereafter, this would imply that only 8.4% of all border crossers remain in the country for more than 10 years. Such a scenario seems inconsistent with other studies showing that the fraction of unauthorized immigrants remaining in the United States for at least 10 years ranges from 34% to 37% between 1995 and 2000 [20].

Upon reflection, however, there is no inherent inconsistency in these scenarios as long as there is heterogeneity in emigration rates between circular migrants and long-term unauthorized residents. Imagine a scenario in which 100 new migrants arrive each year, 80% of whom are circular migrants and 20% of whom are longer-term settlers. Let’s say that the circular migrants emigrate at a rate of 25% per year, while the settlers emigrate at a rate of 2% per year. After many years, approximately 35–40% of the migrant population will have been in the country for 10 or more years. The circular migrants keep circulating so they make up the bulk of the people with less than 10 years in the country. However, the settlers stay, thus building up the population with 10 or more years of U.S. residence. This is very similar to the situation seen in most college towns, where in- and out-migration rates are very high due to the annual circulation of college students, yet there remains a substantial long-term population composed of faculty, support staff, and town residents not connected with the college.

As a final note, we concede that the knowledge base about emigration rates among unauthorized border crossers is weak, so our estimates in Fig 1 are meant to merely illustrate the sensitivity of the estimates rather than provide new estimates of the unauthorized foreign-born population. For example, the MMP provides some helpful information about return migration among unauthorized border crossers, but we still do not know what share of unauthorized border crossers during the 1990s were circular migrants versus settlers. Nevertheless, we do know that the return migration rates used by Fazel-Zarandi, Feinstein and Kaplan pertain to all immigrants rather than unauthorized border crossers, and therefore are not suitable for use in their model. As a result, their model most likely fails to account for a large share of return migration among border crossings during the 1990s.

## Discussion

All models for estimating the unauthorized population are strongly influenced by their assumptions about emigration and mortality. Our analysis of Fazel-Zarandi, Feinstein and Kaplan’s model shows just how sensitive it is to assumptions about emigration. For example, we reviewed their assumptions around emigration of Mexican border crossers during the 1990s and found that much higher emigration could result in a substantially lower unauthorized immigrant population in 2000. Higher emigration would be consistent with a substantial amount of circular migration—as opposed to permanent settlement—among Mexican border crossers during the 1990s. The Mexican Migration Project, a large-scale, well-respected binational research project, provides data supporting higher emigration rates, and several scholars of Mexican migration have noted this circular pattern during the 1990s.

More generally, Fazel-Zarandi, Feinstein and Kaplan provide a very wide range of estimates: from a low or “conservative” estimate of 17 million to a high estimate of 27 million in 2016. Even their conservative estimates imply implausibly high undercounts in the Census of 42% in 2000 and 35% in 2010, the assumptions underlying their method warrant scrutiny. The authors also do not provide benchmarks against any other source such as Mexican Census data.

Fazel-Zarandi, Feinstein and Kaplan have made an important contribution to the demographic study of immigration populations by providing an alternative approach to estimating year-to-year changes in the unauthorized population, particularly for the post 2004 period. The assumptions underlying their method, however, should be better grounded in empirical work for the specific populations and periods being studied. Estimates of return migration, for example, should be based on studies of unauthorized border crossers or visa overstays and not the entire foreign-born population.

Researchers often gather information about populations through U.S. Census-based surveys. Our confidence in these survey-based estimates comes from our confidence in Census field operations, as well as the consistency of these estimates with birth and death records as well as gaps in foreign censuses—specifically the Mexican census—left by their populations’ emigration. Plausible estimates of the any population (not just the unauthorized population) should be consistent with the broader set of data that we have about populations and our understandings of how they grow and change.
